# Evaluating an mHealth App for Health and Well-Being at Work: Mixed-Method Qualitative Study

**DOI:** 10.2196/mhealth.6335

**Published:** 2018-03-28

**Authors:** Elsbeth Marieke de Korte, Noortje Wiezer, Joris H Janssen, Peter Vink, Wessel Kraaij

**Affiliations:** ^1^ Netherlands Organisation for Applied Scientific Research Leiden Netherlands; ^2^ Faculty Industrial Design Engineering Delft University of Technology Delft Netherlands; ^3^ FocusCura Amsterdam Netherlands; ^4^ Netherlands Organisation for Applied Scientific Research Den Haag Netherlands; ^5^ Faculty of Science Leiden Institute of Advanced Computer Science Leiden University Leiden Netherlands

**Keywords:** mHealth, work, qualitative research methods, interview, focus group, technology acceptance, user satisfaction, usability, well-being, prevention

## Abstract

**Background:**

To improve workers’ health and well-being, workplace interventions have been developed, but utilization and reach are unsatisfactory, and effects are small. In recent years, new approaches such as mobile health (mHealth) apps are being developed, but the evidence base is poor. Research is needed to examine its potential and to assess when, where, and for whom mHealth is efficacious in the occupational setting. To develop interventions for workers that actually will be adopted, insight into user satisfaction and technology acceptance is necessary. For this purpose, various qualitative evaluation methods are available.

**Objective:**

The objectives of this study were to gain insight into (1) the opinions and experiences of employees and experts on drivers and barriers using an mHealth app in the working context and (2) the added value of three different qualitative methods that are available to evaluate mHealth apps in a working context: interviews with employees, focus groups with employees, and a focus group with experts.

**Methods:**

Employees of a high-tech company and experts were asked to use an mHealth app for at least 3 weeks before participating in a qualitative evaluation. Twenty-two employees participated in interviews, 15 employees participated in three focus groups, and 6 experts participated in one focus group. Two researchers independently coded, categorized, and analyzed all quotes yielded from these evaluation methods with a codebook using constructs from user satisfaction and technology acceptance theories.

**Results:**

Interviewing employees yielded 785 quotes, focus groups with employees yielded 266 quotes, and the focus group with experts yielded 132 quotes. Overall, participants muted enthusiasm about the app. Combined results from the three evaluation methods showed drivers and barriers for technology, user characteristics, context, privacy, and autonomy. A comparison between the three qualitative methods showed that issues revealed by experts only slightly overlapped with those expressed by employees. In addition, it was seen that the type of evaluation yielded different results.

**Conclusions:**

Findings from this study provide the following recommendations for organizations that are planning to provide mHealth apps to their workers and for developers of mHealth apps: (1) system performance influences adoption and adherence, (2) relevancy and benefits of the mHealth app should be clear to the user and should address users’ characteristics, (3) app should take into account the work context, and (4) employees should be alerted to their right to privacy and use of personal data. Furthermore, a qualitative evaluation of mHealth apps in a work setting might benefit from combining more than one method. Factors to consider when selecting a qualitative research method are the design, development stage, and implementation of the app; the working context in which it is being used; employees’ mental models; practicability; resources; and skills required of experts and users.

## Introduction

### Mobile Health Apps for Health and Well-Being at Work

Workers’ health is of importance to the individual, as well as to the organization in which a person is employed. As healthy workers perform better, workplace interventions are being developed to improve performance, health, and well-being of workers [1-5]. However, research shows that interventions are often not effective, or overall effects are small [3-13]. This calls for exploring new approaches for health and well-being at work.

Mobile and wireless technology (mobile health, mHealth), defined as wireless devices and sensors, including mobile phones worn by persons during their daily activities, is a growing area in supporting health behavior change [14-20].

Various features make mHealth a good candidate for workplace interventions. For example, mobile technology offers the ability to continuously and unobtrusively monitor user’s behavior. Thereby, these technologies can better assess the user’s needs and preferences to deliver context-aware, personalized, adaptive, and anticipatory interventions. In addition, it offers the opportunity to bring interventions into situations where people make decisions about their health and encounter barriers to behavior change. It might also offer cheaper and more convenient interventions with a high penetration and a large reach. Finally, it can support a participative role of users, while enhancing their responsibility over their own health and performance [18-23]. On the other hand, problems have been reported as well, such as quickly declining engagement after usage onset of mHealth apps [24].

### Evidence Base for Mobile Health

Studies on *Web-based interventions* show that they can have positive effects on health knowledge and behavior (eg, [25,26]). These effects also have been shown for Web-based interventions aimed at workers’ health (eg, [27]). However, scientific evidence of *mobile apps* (mHealth) is still limited [28,14].

mHealth apps are being developed and evaluated in a variety of domains such as physical activity (PA) [29-33], obesity [34], and stress management [35]. A lot of these apps have poor or zero evidence base and have not been evaluated with scientific methods [24,36,37]. In recent years, mHealth apps are being developed specifically aimed at risk prevention and healthy behavior in the work setting [38,39], but despite its potential, hardly any research has been published on the content and the effectiveness. Only one study on mobile apps targeting the working population was found, which showed positive effects of a tailored mHealth intervention on PA, snacking behavior, and sleep among airline pilots [40].

Evaluation of mHealth is important, not only to estimate the magnitude of their outcomes but also to ensure they do no harm. Research is not only lacking on health outcomes but also on whether apps actually increase adherence to the behaviors they target and whether apps perform better compared with traditional interventions, either as a stand-alone strategy or integrated within a program [24]. However, technologies can only be effective when they are actually being used by end users. To advance technology design, we therefore need insight into end users’ real-life experiences. Hence, evaluation must involve more than effectiveness evaluation. Testing acceptability and satisfaction of end users plays an essential role as well; this is widely recognized as critical to the success of interactive health applications [17,41]. How is the system used by participants? How well does the system fit into daily (working) lives and context? Which aspects of the system do participants find most helpful or frustrating? How do different components of the system work together? What things do participants wish the system could do? What problems do participants face? Why do participants decline to participate? Why do participants (not) remain engaged over time? [17]. To answer such questions, qualitative methods are needed.

To sum up, despite its great promise, evidence is sparse for mHealth in general [15,17,24] and specifically for risk prevention and healthy behavior at work. Insight is needed whether mobile apps are indeed a powerful medium to deliver interventions at work, a context characterized with its own specific barriers. This is a major scientific knowledge gap and might hamper the adoption of mHealth by the working population. Research is needed to examine its potential and to assess when, where, and for whom mHealth is efficacious, specifically for the working context.

### Evaluating Mobile Health

To study the potential of mHealth apps, quantitative as well as qualitative studies are needed. However, mHealth interventions challenge the way we conduct research. What types of evaluations are appropriate and useful for mHealth apps?

An important challenge is to ensure that an evaluation method matches with the development cycles of technology, which is characterized by a highly iterative process. For instance, to convincingly demonstrate that mHealth apps are effective in changing behavior, often large-scale, long-term studies with control groups such as randomized controlled trials are used [15,17,42-44]. However, in mHealth research, the time it takes to perform high-quality effectiveness studies is critical because technology may be obsolete before a trial is completed. The rapidly evolving nature of both mHealth apps and their uptake means that some components are continuously improved during a trial, though changes to an intervention during an evaluation pose a threat to internal validity [15,43,44].

In addition, it is a challenge to conduct research in an occupational setting [45]. Common examples of challenges are as follows: (1) the organization wants to target all employees with an intervention, although workers might have different needs and goals (eg, some workers suffer from sleeping problems and others need to better balance their work-private life balance); (2) organizations provide only few departments to participate in the research (which might question whether the results represent all employees); (3) the outcomes of interventions depend on the context in which they are delivered, which might be different within an organization (eg, employees performing office tasks or working at an assembly line); and (4) organizations prefer research among their employees to have minimal effect on the daily production processes [45]. The occupational context leads to additional constraints concerning the design of an mHealth intervention and additional constraints concerning the choice of methodologies.

The first step when evaluating novel technologies already starts at the earlier stages of development and consists of gaining a deep understanding of how and why a system is used (or not) [17]. Understanding how technology interacts with other important factors that affect behavior change, such as people’s attitudes and preferences, their relationships, and the context in which they work and live, is critical for the development and adoption of apps [17,45-49].

The focus of this study was to gain insight in users’ real-life experiences of mHealth apps in the working context and the added value of different qualitative methods that might be applied to assess this within this context.

Various qualitative evaluation methods to collect this information are available to apply in one or more stages of an iterative design process [17,46-50]. *Expert-based* methods are commonly used for reasons of practicability, because they are reported to be cheap, fast, and one does not have to recruit users [41,46,47,51]. However, results may not reflect mHealth app use in real practice, as the context in which experts use an app differs from the context of targeted workers. Commonly applied *user-based* methods to gain insight in end users’ real life experiences are focus groups, interviews, surveys, and loggings [41,47,50,52,53]. Focus groups give a quick overview of users’ opinions, and they give insights into the needs of the target group. Part of its value lies in the unexpected findings that can come from free-flowing discussion in the group [50,52,54]. Focus groups require less time burden for an organization than interviews, another frequently adopted method in mHealth evaluation studies [47]. Interviews can be useful to understanding perceptions, opinions, motivation, context of use, and behavior. Generally, compared with the focus group method, interviews take more time but provide deeper insight [54].

### Aim

This study aims to:

- Gain insight in the opinions and experiences of employees and experts on drivers and barriers for using an mHealth app for health and well-being in the working context to develop recommendations for design and implementation
- Gain insight into the added value of different qualitative methods that might be applied within a working context through comparing three different qualitative evaluation methods and assessing whether they yield the same issues evaluating an mHealth app

For this purpose, an mHealth app specifically developed to improve health and well-being of workers at a high-tech company is used as a case study. Three different qualitative methods are used to gain insight in the opinions and experiences of employees and experts on drivers and barriers for using an mHealth app: (1) interviews with end users, (2) focus groups with end users, and (3) focus group with experts. Usability studies have shown that the types of issues revealed by end users’ and experts’ evaluations and by different evaluation strategies only slightly overlap [41,46,47]. Therefore, it is hypothesized that (1) issues revealed by end users’ (employees) and experts’ evaluations only slightly overlap and (2) issues revealed by end users’ interviews and users’ focus groups only slightly overlap. Issues are important topics or points, either neutral, positive, or negative, brought forward by the participants in this study on the use of the mHealth app.

## Methods

### Brightr, a Mobile Health App for Health and Well-Being at Work

**Figure 1:**
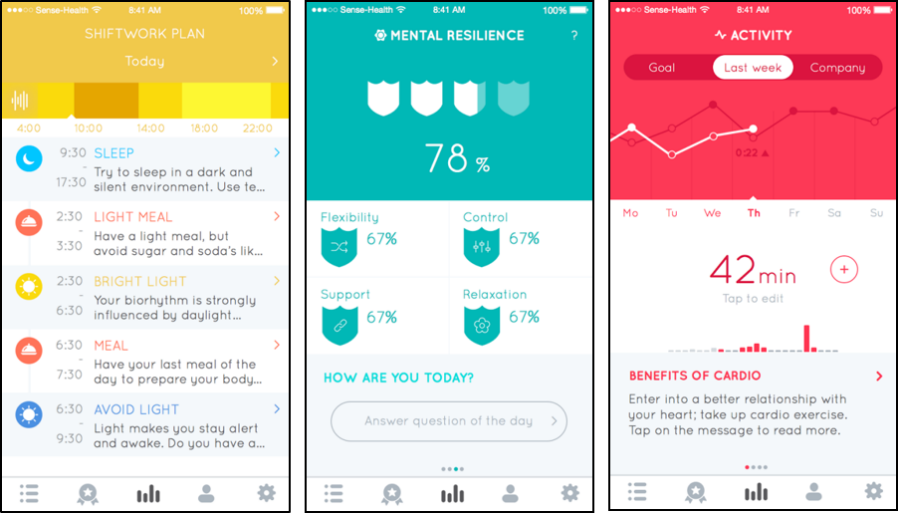
Brightr, examples of the shiftwork, mental resilience, and physical activity modules.

For this study, the Brightr app (version 1.0, Sense Health) was evaluated (Figure 1). Brightr is an mHealth app especially developed for workers at a high tech company to improve their health and well-being. Brightr continuously monitors worker’s behavior, with modules for mental resilience, sleep, PA, nutrition, and shift work. Brightr aims to provide tailored and personalized feedback at the time and place when it matters the most: it offers the possibility to set personal goals that are monitored by short questionnaires (ie, in the mental resilience module) and incorporated sensor data of the mobile phone (ie, to monitor PA and sleep). The collected raw data is then being transformed into real-time human and environmental behavior measurements. On the basis of intelligent algorithms, Brightr provides tailored feedback and advice. In addition, it is possible to compare individual performance with the organization’s average.

### Qualitative Evaluation Methods

This study included end user as well as expert evaluation methods. To get insight in users’ real-life experiences with Brightr, three qualitative methods were used: interviews with end users, focus groups with end users, and a focus group with experts. These methods were applied as is customary in practice, and group sizes of each method were based on what was found in literature. It was planned to conduct between 20 and 25 interviews. In scientific literature, the guideline for the number of interviews is not clear. Some studies show that for an assessment of needs, 10 to 15 interviews will reveal about 80% of the needs [54]. Other studies advice to conduct interviews until saturation is reached and to stop when additional interviews will not yield new information [54,55]. Researchers advice to conduct between 6 and 200 interviews; most of them lie between 5 and 35 [55]. Therefore, aiming to conduct between 20 and 25 interviews was decided to be sufficient to get good results.

Semistandardized telephone interviews were conducted by two experienced interviewers (researcher EK with a background in human factors and ergonomics and researcher NW with a background in social sciences). They worked with an interview guide that contained a list of topics that should be addressed in every interview. After an introduction to the procedures, engagement questions on personal experiences with health and well-being interventions at work were asked. Second, exploration questions were asked on personal experiences with the use of general health and well-being apps and ideas on what kind of features an ideal app for health and well-being at work should have. Then, the Brightr app was evaluated using questions on general impression (eg, “What appeals to you, what not, and why?”), goal (eg, “Could you tell in your own words what the app aims to achieve?”), target group (eg, “For whom do you think this app was developed?”), potential (eg, “What would this app change for you?”), use (eg, “Do you (still) use the app and why (not)?”), outcome expectations (eg, “To what extent does this app fit your needs as a user?”), and information quality (eg, “What do you think about the amount of information to the users?”). The interview ended with general closing questions (eg, “Is there anything else you would like to say about Brightr?”). Before the start of the interview, participants signed an informed consent form. Interviews lasted up to 60 min. The interviews were transcribed verbatim and audiorecorded to fix incomplete data during transcription.

The aim was to plan focus groups with a recommended size of 6 to 8 participants [54]. Three focus groups were conducted with end users (duration 90 min) at their company and one with experts (duration 120 min, at the research institute of EK and NW) by two experienced focus group facilitators: researchers EK and NW. Both researchers facilitated two focus groups and transcribed verbatim two times during the group discussions. The facilitator used a focus group guide that covered the same topics as the interview guide. Before the start of the focus group, participants signed an informed consent form. The focus group discussions were also audiorecorded to fix incomplete data during transcription.

### Participants

Brightr was offered to all employees of a high tech company, and they were able to download the app on a voluntary basis. Before recruitment for the evaluation study started, employees had the opportunity to use the app for at least 3 weeks. Employees were recruited for this study by a message on the company website and by messages on the information screens in the hallways that contained a link to the message on the company website. The message contained information on the aim, the setup, and data privacy of the study. To get insight in reasons for declining to use Brightr, employees were asked to follow a link in case they stopped using Brightr. This link directed to a questionnaire (Survalyzer) with two questions on the reasons for not using Brightr and on conditions or situations under which they would like to use an app such as Brightr. Employees using Brightr who were interested to participate in the study were asked to follow a link to another questionnaire (Survalyzer). It contained questions on gender, age, function group (operations and order fulfillment, sales and customer support, development and engineering, or support staff), hours working per week (flexible contract, 24 hours or less, 24-32 hours, or more than 32 hours), work experience at the company, and email address. This information was used to plan homogenous interview groups and focus groups. The email addresses were used to contact the participants to plan interviews and focus groups. Participants who declined an invitation for a focus group, for example, because the focus group was planned on an unfavorable timeslot for them, were asked to participate in an interview.

The experts were recruited by sending them an email with an invitation to participate in the study along with information about the aim and the setup of the study. They were asked to use the Brightr app for 3 weeks before they participated in a focus group. A total of 15 experts were recruited among the personal networks of two researchers (EK and NW) and consisted of behavioral scientists, psychologists, ergonomists, designers, human-computer interaction researchers, and policy makers. Upon acceptance of the invitation, experts received the Brightr app. To ensure a psychologically safe atmosphere, in which participants felt no barriers to speak freely, developers of the Brightr app (eg, researcher JJ) were excluded from the expert focus group.

### Analysis

Qualitative data analysis was aimed to assess and compare issues addressed by end users in interviews and focus groups and by experts in a focus group. Data were collected from March 2015 to July 2015.

A codebook was constructed to analyze all transcripts. The codebook uses constructs from user satisfaction and technology acceptance models to understand and evaluate factors explaining users’ perception about information systems to assess actual usage of these systems. Definitions used in the codebook of this study are adapted from the framework of Wixom and Todd [48], Bailey and Pearson [56], and Vosbergen et al [46] and specified further to the mHealth app that was used in this study. The final codebook can be found in Multimedia Appendix 1.

Data were categorized according to the following scheme: domain from the codebook, topic from the codebook, and whether the quote was positive, negative, neutral, or a recommendation, comparable to the analysis performed by Vosbergen et al [46]. In case a quote addressed multiple topics, it was categorized multiple times using different codes.

Two researchers (EK and NW) independently coded transcripts. After each transcript, they resolved discrepancies in discussion meetings up to the point they reached 80% matching codes, which was at the sixth transcript. The remaining transcripts were then evenly divided between researchers. Coded transcripts were included in Excel (Microsoft). Descriptive statistics were used to assess whether the three different qualitative analyses yielded the same issues evaluating Brightr and to gain insight in experiences and opinions that were obtained in general on drivers and barriers using Brightr in the working context.

## Results

### Nonparticipants

**Figure 2:**
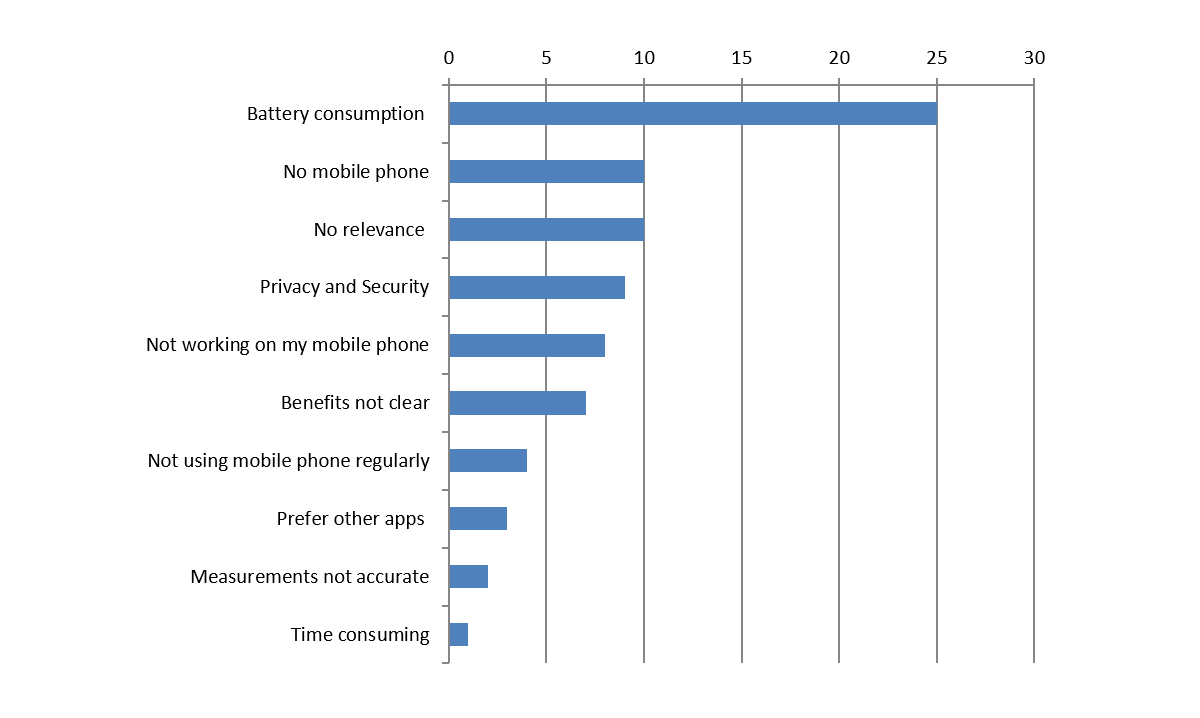
Main reasons (number of times mentioned) of 79 employees on why they declined to use Brightr and therefore, did not participate in the study.

In the recruitment phase, 79 employees who declined to use Brightr filled in the two questions in Survalyzer on reasons for not using Brightr and conditions under which they would consider using an app such as Brightr. This group consisted of employees who never started using Brightr and employees who stopped using Brightr after a short period of time. How many employees never started to use Brightr is not known, nor is it known how long employees used Brightr before they stopped using it. This may have varied between just having a look at the app to using it for about 3 weeks. Figure 2 shows the main reasons of employees for not using Brightr. The most important reasons for not starting or quitting with Brightr were the large battery consumption of the app, not having a mobile phone, and the app had no relevance for the person. A total of 51 employees indicated that they would consider using Brightr under certain conditions. Most important conditions to consider using Brightr were improvements in battery use, clearer relevance for the user, and when the app would function on their mobile phone. A total of 28 employees would not consider using Brightr at all.

### Participants

Reminders to participate in the study were sent twice via a pop-up message in the Brightr app to all users. After recruitment, 59 employees agreed to participate in the study. They received an invitation to plan an appointment for an interview or focus group. With 41 employees, an interview or focus group was planned. With 18 employees, it was not possible to plan an appointment because they did not respond to email messages or were absent from work because of sickness or vacation. Due to difficulties to recruit employees for the study, it was not possible to create homogeneous groups for interviews and focus groups.

With 22 employees, interviews were planned. The three focus groups with employees consisted of 4, 5, and 6 participants, respectively. Six more people were planned to participate in a focus group but declined, and 2 of them participated in an interview later on. Employee characteristics are shown in Table 1. Six experts (1 male, 5 female) participated in the focus group for experts. All participants obtained a university MSc and/or PhD in artificial intelligence, computer science, public administration, social sciences, or human movement sciences. They had expertise in the areas of behavior change, machine learning, big data and sensor data analysis, work-related stress, shiftwork, sustainable employability, electronic health or mHealth, mental resilience, PA, and intervention methods. All of the experts used Brightr for 3 weeks.

### Issues Yielded With Three Qualitative Methods

Interviewing employees yielded 785 quotes, focus groups with employees yielded 266 quotes, and the focus group with experts yielded 132 quotes (Table 2).

### Overview of Similarities and Differences per Domain

Table 3 gives an overview of issues (neutral, positive, or negative) per domain. Interviews with employees yielded the highest percentage of issues within the domain of usefulness (25.5%, 200/785), followed by information quality (23.3%, 183/785). Focus groups with employees yielded also the most issues in the usefulness domain (27.4%, 73/266), which was followed by system quality (21.1%, 56/266). The focus group with experts yielded most issues in the system quality domain (23.5%, 31/132), followed by usefulness (22.7%, 30/132). In general, least issues were yielded on service quality.

**Table 1:** Employee characteristics.

Characteristics		Interviews	Focus groups
Number of employees, n		22	15
Years working at company, mean (SD)		6.6 (5.6)	10.4 (6.6)
Age (years), mean (SD)		39.0 (8.7)	45.2 (11.1)
**Gender, n**			
	Male	17	13
	Female	5	2
**Function, n**			
	Operations and order fulfillment	7	5
	Sales and customer support	1	1
	Development and engineering	9	5
	Support function	5	4
**Working hours, n**			
	Flexible or 0 hours	0	0
	24 hours or less	1	0
	24-32 hours	2	3
	More than 32 hours	19	12

**Table 2:** Number of participants in interviews and focus groups and number of quotes that were yielded with three different qualitative methods.

Qualitative method characteristics	Interviews employees, n	Focus groups employees, n	Focus group experts, n
Number of participants	22	15	6
Number of quotes	785	266	132

**Table 3:** Overview of issues per domain (number and percentage).

Domain	Issues, n (%)		
	Interviews employees	Focus groups employees	Focus group experts
System quality	98 (12.5)	56 (21.1)	31 (23.5)
Information quality	183 (23.3)	47 (17.7)	19 (14.4)
Service quality	8 (1.0)	3 (1.1)	0 (0.0)
Usefulness	200 (25.5)	73 (27.4)	30 (22.7)
Ease of use	48 (6.1)	8 (3.0)	11 (8.3)
Outcome expectations	126 (16.1)	39 (14.7)	17 (12.9)
Organizational factors	121 (15.4)	40 (15.0)	24 (18.2)

### Overview of the Value of Issues per Domain

Table 4 shows the number and percentage of positive, negative, or neutral issues and recommendations per domain.

Interviews yielded mostly recommendations within the domain of information quality and organizational factors. This method generated mainly negative issues in the domains of system quality, usefulness, ease of use, and outcome expectations. In contrast to both other methods, employee focus groups yielded mostly neutral issues within two domains: service quality and organizational factors. This method also generated mainly positive issues in the usefulness domain. Employee focus groups only yielded mostly recommendations in the domain of outcome expectations. This method generated mostly negative issues in the domains of system quality, information quality, and ease of use.

Experts gave mostly recommendations within the domains of system quality, outcome expectations, and organizational factors. No issues were yielded within the domain of service quality. In all other domains, experts mainly generated negative issues.

### Similarities and Differences per Topic

In Table 5 for each domain the underlying topics that were yielded by the employees (interviews and focus groups) and experts (focus group) are shown. An overview of illustrative examples of quotes is shown in Multimedia Appendix 2.

#### System Quality

Within the domain of system quality, issues of experts mostly focused on the topic “tailoring” (42%, 13/31), about 3 to 4 times as many as addressed by employees in respective interviews and focus groups (Table 5.). Experts especially stress the importance to tailor the app to the goals of the user and to personalize behavior change techniques, preferably using learning algorithms. Employees typically recommend tailoring the app to age, condition, and functioning type (ie, heavy work or desk work).

Employees mostly focused on the topic “performance of the system” (55/98, 56% and 21/56, 38% of the quotes in interviews and focus groups, respectively), whereas only 7% (2/7) of the quotes of experts were about this topic. Employees’ quotes mainly focused on the high battery use; this was often a reason for quitting the use of Brightr. None of the experts made a quote on batteries. The system not working properly was another important issue on system performance for employees; it was either working too slow or having bugs.

In addition, in both focus groups, “time lines” was the second most addressed topic, 21% (12/98) and 23% (7/31) for employees and experts, respectively, almost twice as many as in the interviews (10%, 10/98). Issues on time lines mainly addressed the moments people use the app. An employee from the focus group stated:

When I receive a message, I take a look at the app. However, I take a look less often now, mostly in the evening or when I am at the toilet.

Neutral quote

#### Information Quality

For the information quality domain, about one-third of the issues were yielded on the topic “content” of the app; this was similar for all types of methods. Employees mainly addressed the topics they would like to see in the app, for instance, food, sports, or work-rest schedules. An interviewed employee gave the following recommendation (see Multimedia Appendix 2):

I would like more information about food, what you should eat. Shift workers have to eat very fast at times (and therefore, the choices are not always healthy). I would like tips about food that is healthy and that you can eat fast.

Recommendation

Experts were mainly positive about the different aspects that were addressed by the app and gave recommendations on the content of the feedback:

I think of an app that shows the effects of your behavior, for example to show visually “what you have done now leads to this effect.”

Recommendation

Next to the topic “content,” interviews with employees yielded much issues on “format” (45/183, 24.6%; most employees liked the look and feel of the app). For both focus groups, “accuracy” of the app was an important topic (16/47, 34% with employees and 5/19, 26% with experts). Often, people doubted accuracy of the sleep measurements.

**Table 4:** Number and percentage of positive (+), negative (−), and neutral (0) issues or recommendations (R) within each domain.

Domain and value		Issues within domain, n (%)		
		Interviews employees	Focus groups employees	Focus group experts
**System quality**				
	+^a^	11 (11)	2 (4)	0 (0)
	−^b^	51 (52)	31 (55)	5 (16)
	0^c^	11 (11)	9 (16)	8 (26)
	R^d^	25 (26)	14 (25)	18 (58)
**Information quality**				
	+	43 (23.5)	6 (13)	6 (32)
	−	59 (32.2)	24 (51)	10 (53)
	0	10 (5.5)	1 (2)	0 (0)
	R	71 (38.8)	16 (34)	3 (16)
**Service quality**				
	+	3 (38)	1 (33)	0 (0)
	−	2 (25)	0 (0)	0 (0)
	0	0 (0)	2 (67)	0 (0)
	R	3 (38)	0 (0)	0 (0)
**Usefulness**				
	+	53 (26.5)	29 (40)	6 (20)
	−	70 (35.0)	16 (22)	14 (47)
	0	39 (19.5)	7 (10)	7 (23)
	R	38 (19.0)	21 (29)	3 (10)
**Ease of use**				
	+	20 (42)	0 (0)	0 (0)
	−	21 (44)	6 (75)	7 (64)
	0	0 (0)	2 (25)	2 (18)
	R	7 (15)	0 (0)	2 (18)
**Outcome expectations**				
	+	32 (25.4)	8 (21)	1 (6)
	−	55 (43.7)	11 (28)	3 (18)
	0	12 (9.5)	6 (15)	5 (29)
	R	27 (21.4)	14 (36)	8 (47)
**Organizational factors**				
	+	13 (10.7)	4 (10)	0 (0)
	−	27 (22.3)	8 (20)	2 (8)
	0	37 (30.6)	15 (38)	3 (13)
	R	44 (36.4)	13 (33)	19 (79)

^a^+ symbol signifies positive.

^b^− symbol signifies negative.

^c^0 signifies neutral.

^d^R signifies recommendations.

**Table 5:** Topics of issues (number and percentage within domain).

Domain and Topic		Issues within domain, n (%)		
		Interviews employees	Focus groups employees	Focus group experts
**System quality**					
	Accessibility	0 (0)	0 (0)	0 (0)
	Time lines (responsiveness)	10 (10)	12 (21)	7 (23)
	Flexibility	10 (10)	5 (9)	5 (16)
	Integration	5 (5)	8 (14)	2 (7)
	Efficiency	5 (5)	1 (2)	0 (0)
	Tailoring	13 (13)	6 (11)	13 (42)
	Language	0 (0)	1 (2)	1 (3)
	Errors or error prevention	0 (0)	2 (4)	1 (3)
	Performance	55 (56)	21 (38)	2 (7)
**Information quality**					
	Accuracy	34 (18.6)	16 (34)	5 (26)
	Precision	5 (2.7)	0 (0)	0 (0)
	Reliability	1 (0.5)	4 (9)	1 (5)
	Currency	5 (2.7)	2 (4)	0 (0)
	Completeness	15 (8.2)	4 (9)	2 (11)
	Format	45 (24.6)	4 (9)	3 (16)
	Volume	9 (4.9)	1 (2)	2 (11)
	Content	69 (37.7)	16 (34)	6 (32)
	Visibility of system status	0 (0.0)	0 (0)	0 (0)
**Service quality**					
	Relationship with app provider	2 (25)	0 (0)	0 (0)
	Communication with app provider	2 (25)	2 (67)	0 (0)
	Technical competence of app provider	1 (13)	0 (0)	0 (0)
	Attitude of app provider	0 (0)	0 (0)	0 (0)
	Schedule of products or services	2 (25)	0 (0)	0 (0)
	Processing of change requests	0 (0)	0 (0)	0 (0)
	Response time	0 (0)	0 (0)	0 (0)
	Means of input with app provider	1 (13)	1 (33)	0 (0)
**Usefulness**					
	Usefulness	14 (7.0)	18 (25)	4 (13)
	Relevancy	110 (55.0)	42 (58)	13 (43)
	Adherence	76 (38.0)	13 (18)	13 (43)
**Ease of use**					
	User-friendly	21 (44)	3 (38)	1 (9)
	Easy to use	8 (17)	0 (0)	0 (0)
	Learnability	18 (38)	5 (63)	10 (91)
	Memorability	1 (2)	0 (0)	0 (0)
**Outcome expectations**					
	Expectations	30 (23.8)	5 (13)	0 (0)
	Understanding of system	4 (3.2)	0 (0)	0 (0)
	Confidence in the system	14 (11.1)	5 (13)	1 (6)
	Feelings of participation	2 (1.6)	5 (13)	2 (12)
	Feelings of control	23 (18.3)	11 (28)	6 (35)
	Degree of training	0 (0.0)	0 (0)	1 (6)
	Accuracy	12 (9.5)	4 (10)	0 (0)
	Health and performance effects	41 (32.5)	9 (23)	7 (41)
**Organizational factors**					
	Management involvement	6 (5.0)	4 (10)	5 (21)
	Organizational competition	5 (4.1)	3 (8)	6 (25)
	Security of data	39 (32.2)	15 (38)	7 (29)
	Documentation	0 (0.0)	4 (10)	3 (13)
	Timing	22 (18.2)	6 (15)	0 (0)
	Communication	49 (40.5)	8 (20)	3 (13)

#### Service Quality

Service quality was the least mentioned domain. Experts did not mention this domain and its topics at all. Interviews, as well as focus groups with employees, yielded the topics ‘communication with the app provider and “means of input with app provider.” In addition, in interviews, extra topics were addressed compared with the focus groups with employees: relationship with app provider, technical competence of app provider, and schedule of products and services.

#### Usefulness

Within the domain of usefulness, “relevancy” was the most addressed topic for each of the evaluation methods: 55.0% (110/200) of the quotes in employee interviews, 58% (42/73) in employee focus groups, and 43% (13/30) in expert focus groups.

All groups mainly focused on the extent to which the app or different aspects of the app helped to solve their problems (eg, sleep, stress, and healthy eating) or whether it addressed interests (eg, sports and food). An illustrative quote from an employee interview is as follows:

The best part, for me, is the shiftwork part (I work morning, evening, night shift). Since I try to follow the advices about maintaining a healthy lifestyle and working with shift hours. It helped me to keep down the stress in my body. I felt that I could focus better on the task during the daily (nightly) work.

Positive quote

An employee in a focus group stated:

The mental resilience part is doing absolutely nothing for me. I often think: for what reason am I doing this? If you are doing well, it has no added value.

Negative quote

An example of an expert quote is as follows:

Mental resilience also triggered...well, it yielded only frustration, I did not receive any tips.

Negative quote

For employees in the focus groups, “usefulness” was the second most addressed topic (25%, 18/73). One of the employees expressed:

It triggers to do things better in your behavior. The fact that I saw that I pretty quickly reached my physical activity goals was good, to see that it was not a problem for me.

Positive quote

For the other two groups, this was “adherence” (76/200, 38.0% for interviewed employees, 13/30, 43% for experts). Results showed that employees quit using the app mainly because of system failures, extensive battery use, or absence of relevancy, while push messages stimulate the use. Overall, many employees mentioned a decrease in use over time. Experts also mentioned system failures as a reason for attrition and stressed the importance of addressing user motivation.

#### Ease of Use

Within the domain “ease of use,” employees as well as experts experienced problems with discovering certain content and features of the app. One expert stated:

I found out only after a week that there was more than just physical activity. I swiped once accidentally and there they were: all sorts of modules!

Neutral quote

Interviewed employees focused mainly on the topic “user friendliness” (21/48, 44% of the issues, concerning positive as well as negative user experiences), followed by “learnability” (38%, 18/48). Results were opposite for both focus groups: the topic “learnability” was most important for employees and experts in focus groups (5/8, 63% and 10/11, 91%, respectively). In contrast with interviews, within both focus groups, the topics “ease of use” and “memorability” were not mentioned at all.

#### Outcome Expectations

The topic “health and performance effects” was mentioned most often within the domain of outcome expectations; for interviewed employees and experts, it was the number one most addressed topic (41/126, 32.5% and 7/17, 41%, respectively), and for employees in focus groups, it was the second most addressed topic (23%, 9/39). The opinions on health and performance effects of interviewed employees were mixed: some declared that the app actually helped them to behave healthier, some think that an app such as Brightr is able to raise at least awareness, and others have doubts about the ability to change behavior or affect health. One of the interviewed employees stated:

There are many different kinds of workers in our company, some need physical activity advice (eg, they lift weights a lot at work), others have to exercise more (eg, sitting at desk to much). An app can help them to become aware. Goal of such an app is to try to get people think whether they are in balance. Do they have sufficient activity? I think it is possible that an app could help to reach goals. Reaching some goals must be possible.

Positive quote

Employees in focus groups showed similar opinions. Experts also showed mixed opinions about health and performance effects, but they focused more on different types of intervention functions apps might have, such as raise awareness, provide insight, give instruction, or change behavior and whether Brightr was able to do that (some agreed, some disagreed). For both focus groups, feelings of control appeared to be the second most important topic. Experts mainly stressed the importance of giving control to app users, for instance to set personal goals. They also discussed whether a user is able to decide for himself what he needs from a health perspective. Although employees also mentioned the significance of user autonomy, they were more focused on the possibilities to adjust missing data (eg, when they did not carry their mobile phone with them) or incorrect data (eg, app measuring walking instead of cycling).

#### Organizational Factors

“Organizational factors” is an important domain to assess issues that influence uptake and implementation of mHealth apps in the working context. For the interviewed employees, “communication” was the most addressed topic within the domain of organizational factors (49/121, 40.5% of issues). It mainly addressed the way the app was implemented within their organization and how this was influenced by the relationship between employer and employee. Often they focused on whether management should play a role in implementation (management setting an example) or not (an organization should keep a certain distance when it comes to such personal data). For focus groups, security of data was most important; it was the second most addressed topic for the interviewed employees. Employees in interviews as well as in focus groups showed mixed opinions on data privacy and security. For some it is an important issue, for others it is not. Some employees mentioned that giving feedback to managers on an aggregated level might provide useful information for management. Experts mostly stressed the importance of being very concise and transparent on what happens to the data. “Management involvement” and “organizational competition” (congruence between assessment and feedback provided by the system and an external health professional or system [eg, coach, other app, and other system]) were least addressed by employees in interviews and focus groups, but gained much more attention by the experts. Experts mainly recommended organizations to embed an app such as Brightr in a bigger health or vitality program:

App should be a part of a bigger program, in terms of intervention. It is supportive within an intervention.

Recommendation

## Discussion

### Drivers and Barriers Using Mobile Health in the Working Context

The findings in this study suggest a number of valued characteristics, as well as challenges that organizations might consider for mHealth app and implementation and developers might use for design to enhance user satisfaction and technology acceptance. Overall, participants muted enthusiasm about the app. This is in line with the research of Dennison et al [20] who found similar results in their qualitative study on mobile phone apps supporting health behavior change among young adults. However, Dennison et al [20] found context sensing and social interaction features to be unnecessary and off-putting. This is in contrast with our study in which participants recommended to develop these features in future versions of the app, for example, the interest to compare personal data with organizational means or tailoring the personal advice to the shift work schedules. Apparently, to take context into account is very important for the application of mHealth in the working context but might be less important for other contexts of use. Combining results from the three evaluation methods that we used in our study, results show the following recommendations when designing mHealth apps for health and well-being at work.

#### Technology

System failures or poor performance (eg, high battery use) does influence adoption and adherence to mHealth apps negatively. Accuracy of measurements largely influences the confidence of users in the app and thereby influences its use. Accuracy (actual as well as perceived) but also the quality of the advice largely influences the possibility to reach behavior change and in line with that health and performance effects. It should therefore be based on solid evidence.

#### User Characteristics

Relevancy and benefits of the app should be made clear to the (potential) user, within the app itself, as well as in communication guiding the implementation of the app. Furthermore, the app has to address users’ characteristics (age, condition, health, function, and [work] activities), motivation, and needs (eg, health [risks] and well-being). A next step in developing apps should aim at using machine learning and learning algorithms to tailor the app to user characteristics automatically. A point of attention is giving users much autonomy, for instance, in ways to use the app, setting and adjusting goals, and when and how to receive feedback. Giving users autonomy in what they should need from a health point of view should be considered carefully, as users might not be aware of their health behaviors.

#### Context

It is very important to take into account the work context in which the app is being used. For instance, sometimes it is not possible to use a mobile phone in specific work contexts (eg, clean rooms), which affects the accuracy of the measurements. A suggestion might be to combine mobile phone apps with a wearable sensor that is possible to wear continuously in all (work) contexts. This suggestion is in line with Coursaris and Kim [57] who suggest to design interfaces and apps that fit particular contextual settings, while being flexible to accommodate others: “focus beyond the interface when designing applications” [57]. Furthermore, implementation plays a large role in the adoption and use of an app; this should thus be planned carefully, of which considering how and to what extent the management should be involved is an important factor. Experts suggest to embed such apps within a larger intervention to improve opportunities for success.

#### Privacy and Autonomy

Results showed that for different end users privacy was either not an issue or an important issue. Van Lieshout et al [58] give some implications for dealing with apps that are offered by employers to their employees: an app offered by the employer always has to be used on a voluntary basis. Employees always should be alerted to their right to privacy and before apps are offered, and employees must be properly informed. In addition, within an organization, it should be very clear what happens to the data. Moreover, users should be given autonomy in deciding what happens to the data; various tools offer guidelines, for instance, Privacy by Design or Privacy Impact assessment [58,59].

### Applying Qualitative Methods Within a Working Context

Although studies have used qualitative evaluation methods in testing mobile apps [47,51,60-62] or compared qualitative evaluation methods in other apps, such as testing websites (eg, [46,63-67]), to our knowledge, this study was the first to assess whether different qualitative methods yield the same or different issues when testing an mHealth app for health and well-being at work.

The results of this study showed that issues revealed by experts only slightly overlapped with those expressed by employees. In addition, it was seen that interviews yielded different results compared with those from focus groups. These results are in line with conclusions from other studies comparing different qualitative evaluation methods: different methods identify unique issues, often more than common issues (eg, [47,51,63]).

Our study showed that the *type of evaluators* influences the kinds of issues an evaluation yields. The differences were seen in the attention that was given to the higher level of domains, as well as on the underlying topics that were addressed. For instance, the usefulness domain was given most attention by employees, whereas experts gave most attention to system quality. Moreover, differences were found in the values of remarks: positive, negative, neutral, or a recommendation. Although it was expected that experts would give many recommendations for improvement, they also yielded many negative remarks. Finally, analyzing the remarks itself, it was seen that even similar coded remarks were different in nature.

Employees gave insight into immediate practical experiences. The degree to which the app meets the needs of the employees and addressed their problems or interests is important for starting or continuing the use of the app. Furthermore, they described what motivated them to use the app, what prevented them from using it (such as system failures), and whether privacy of data played a role in using the app. This is in line with the findings of Vosbergen et al [46], but less in line with Lathan et al [67], who examined a Web-based system and found that users were mainly interested in efficient and effective use of the system. Results of this study are also in line with the work of Nielsen and Randall [68] on evaluating organizational-level interventions, who argue that insight into employee experiences is important to match an intervention to identified problems and to match it with the specific individual working context.

In this study, experts were more focused on higher level issues, building on their knowledge of theories and models, and using approaches derived from scientific knowledge and expertise. This is in line with Vosbergen et al [46] and Jaspers [41]. The experts in this study emphasized quality and evidence base of the information and ways to enhance adoption and continuous use by employees: accuracy of measurements, tailoring the app to user needs and providing users with autonomy (within certain boundaries), addressing user motivation, implementation of the app within the organization, and embedding in larger health or vitality programs. When implementing an app such as Brightr, they stressed that the intervention function of an app should be clear: raising awareness, providing insights, giving instructions, or changing behavior, as this influences the design of the app. Finally, they stressed the importance of transparency of data. According to Nielsen and Randall [68], who developed a model for evaluating organizational-level interventions, expert opinions are important as they are focused on the broader context of interventions and the use of theories. They understand the links between work and health and the underlying mechanisms, which is necessary to develop and implement effective interventions such as mHealth apps.

Tan et al [63] conclude that methods using experts or using end users complement each other and that neither method could be replaced by the other. They suggest using experts especially in the early design stages of development as they address user issues on a higher level, whereas user testing should be conducted in later stages as it needs a well-developed test bed. Vosbergen et al [46] concluded that an evaluation cannot be performed without end users, and the results of our study subscribe these conclusions. Vermeeren et al [51] and Adams and Cox [69] describe the importance of recruiting experts with required expertise, preferably with the right domain expertise.

Our study also revealed that the *type of evaluation* influences the kinds of issues an evaluation yields: issues addressed by employees in interviews differed from the issues addressed by employees in focus groups. This was seen in the attention that was given to certain domains, the values of the remarks within the domains, as well as the topics within each domain. Zapata et al [47] found four different evaluation methods in their systematic review that were used in mHealth evaluations: questionnaires, interviews, logs, and “think out loud” method. Questionnaires were the most applied method, followed by interviews. They did not find studies that used focus groups as an evaluation method for mHealth apps. This study shows that conducting focus groups for evaluating mHealth apps in the working context provides valuable information.

Often, a method is chosen on the basis of practicability [51,69]. Focus groups seem efficient because it gives a quick overview of opinions of multiple users at the same time [54]. Conducting interviews is a time-consuming process but offers the possibility of obtaining detailed and thorough information compared with, for instance, a questionnaire [69]. Some issues are for ethical and privacy reasons better dealt with in interviews, whereas a focus group will allow for easier reflection on common experiences [69]. This study did not confirm the idea that interviews lead to deeper insights or more detailed information as Van Boeijen et al [54] state; in this study, differences were found in the domains and underlying topics that were addressed, and results seemed of similar level of detail. Nor did this study confirm that ethical and privacy issues were better dealt with in interviews compared with focus groups [69]. In both settings, interviews and focus groups, employees in this study felt free to speak. From a practical point of view, our study showed that conducting focus groups is a more efficient qualitative method to evaluate an mHealth app than conducting interviews. Although both evaluation methods address overlapping issues, a focus group might offer more information on common or different experiences, for example, on factors such as (middle) management support, employee support, participation, information, and communication. In interviews, detailed individual experiences might have a more prominent role, such as the individual working conditions and individual factors such as readiness for change, perceptions, and appraisals.

### Limitations

Several limitations of our study have to be discussed. Due to difficulties to recruit employees for the study, it was not possible to create homogeneous groups for interviews and focus groups. Results on the analysis of the questionnaire data of nonparticipants showed that our final group of employees probably has been biased. Within our sample of employees, individuals who were more motivated to respond (for instance, because they have strong opinions on the mHealth app) might have been overrepresented, as we used a self-selection protocol during recruitment.

Furthermore, although the total number of participants is larger than in most studies on user experiences, all three evaluator groups differed in size: 22 employees were interviewed, 15 employees participated in three focus groups, and 6 experts participated in one focus group. As a consequence, large differences between the number of remarks yielded by each method were found. To compare between methods, we therefore used percentages.

In addition, the three methods differed in the evaluation technique and the instructions that were given. These variations influenced results and made it difficult to examine the causes of the differences that were found between the three evaluation methods. However, the goal was to compare three different methods in the way they are commonly used in practice, not to compare them in an experimental setting with controlled variations. For this purpose, three methods were compared using one case study with the Brightr app to make a systematic comparison of methods; the study would have to be repeated in more settings.

Moreover, an early version of the Brightr app was used while conducting the study. On one hand, this might have skewed the responses to focus more on system quality and accuracy as compared with an app that has been developed further. On the other hand, this might have provided extra points of feedback that might otherwise not have been compared between qualitative methods.

Finally, a limitation of our study lies within the rating process. For time efficiency reasons, 2 raters independently coded remarks and resolved discrepancies in discussion meetings up to the point they reached 80% matching codes, at the sixth transcript. The remaining transcripts were then evenly divided between researchers. Although this procedure has been followed to reach a certain degree of reliability, no interrater reliability tests have been performed, and raters might have used different interpretations in rating the remaining transcripts.

### Conclusions

Findings in this study provide the following recommendations for organizations planning to provide mHealth apps to their workers, as well as for developers of mHealth apps: (1) system performance influences adoption and adherence, (2) relevancy and benefits of the mHealth app should be clear to the user and should address users’ characteristics, (3) app should take into account the work context, and (4) employees should be alerted to their right to privacy and use of personal data.

When considering which qualitative method to apply in a work setting, findings in this study showed that the type of evaluators as well as type of evaluation method influences which kinds of issues will be generated. The results revealed that different evaluation methods are complementary and therefore, evaluation processes might advantage from combining more than one method, which is also concluded by others [47,51,62-64]. Factors to consider when selecting methods for a qualitative evaluation of mHealth apps in the occupational setting are as follows: required information on the design and implementation of the mHealth app, the working contexts in which it is being used and participants’ mental models on the mHealth app and context; the development stage of the app; practicability; resources; and skills required of experts and/ or users.

However, more scientific insight on these issues is still necessary. Furthermore, which methods work best in what situation and which methods work well together are still questions under research.

## Appendix

Multimedia Appendix 1 Codebook.

Multimedia Appendix 2 Illustrative quotes.

## Abbreviations

mHealth: mobile health
PA: physical activity
