# Supplementary material for: Evaluating an mHealth App for Health and Well-Being at Work: Mixed-Method Qualitative Study
Source: JMIR Mhealth Uhealth. 2018 Mar 28;6(3):e72. doi: 10.2196/mhealth.6335 (PMC5895922; doi:10.2196/mhealth.6335)
Supplement: Multimedia Appendix 1 [file mhealth_v6i3e72_app1.pdf]

Multimedia Appendix 1. Codebook

This is a Multimedia Appendix to a full manuscript published in the JMIR mHealth and uHealth. For full copyright and citation information see http://mhealth.jmir.org/0000/0/e0/ doi:10.2196/mhealth.6335.

The codebook is an adapted version of the codebook used by Vosbergen et al (2014) who used the domains and topics described by Wixom and Todd (2005) in their study on evaluating a web-based health risk assessment tool. Definitions are adapted from the framework of Wixom and Todd (2005), Bailey and Pearson (1983) and Vosbergen et al. (2014) and further specified further to the mHealth application that was used in the current study. Some topics that were part of the framework of Wixom and Todd (2005) or part of the codebook of Vosbergen et al (2014) are not described. New topics were added in case the researchers felt it was missing, these were performance (system quality), content, visibility of system status (information quality), adherence (usefulness).

Table. Definitions of domains and topics of the codebook used to categorize all remarks of employees and experts

**Domain**	**Topic**	**Definition**
**System quality**		**Users' perceived quality of the app**
	Accessibility	The degree to which the system is accessible to its users
	Timelines (responsiveness)	The availability of the system's output at a time suitable for its use
	Flexibility	The capacity of the system to change or adapt in response to new conditions, demands, or circumstances
	Integration	The ability of systems to communicate/ transmit data between systems servicing different functional areas, e.g. to link together different components of the app to act as a coordinated whole
	Efficiency	The rate or speed at which the system enables users to accurately and successfully complete a task
	Tailoring	The ability of the system to take user characteristics into account
	Language	The set of vocabulary, syntax, and grammatical rules used to interact with the system
	Errors / error prevention	The methods and policies governing correction and rerun of incorrect system output
	Performance	Technical performance of the hardware and the software
**Information quality**		**Users' perceived quality of the information given by the app**
	Accuracy	Users' perception that the information is correct
	Precision	The variability of the output information from that which it purports to measure
	Reliability	The consistency and dependability of the output information
	Currency	The age of the output information
	Completeness	The degree to which the app provides all information perceived as necessary by the user
	Format	The layout and display of the information throughout the entire app

	Volume	The amount of information conveyed to users
	Content	The content of the information provided
	Visibility of system status	The degree to which the system keeps the users informed about what is going on, through appropriate feedback within reasonable time.
**Service quality**		**Users' perceived quality of the service delivered by the professionals associated with the app**
	Relationship with app provider	The method and manner of interactions between users and app provider
	Communication with app provider	The way information is exchanged among users and app provider
	Technical competence of app provider	The skills and expertise of the app provider
	Attitude of app provider	The way users perceive the attitude of the app provider towards users and their health experiences
	Schedule of products or services	The timetable for system output, services, and procedures
	Processing of change requests	The manner, methods, and required time the staff respond to users' requests
	Response time	The time between users' requests for service or action and response to these requests
	Means of input with app provider	The method and medium by which users receive services from app provider and/or the system and the perceived usefulness of this service
**Usefulness**		**General usefulness of the app for its users**
	Usefulness	The extent to which the app actually helps to solve users' problems
	Relevancy	The degree of congruence between users' needs and requirements and what the app provides
	Adherence	The extent to which the app stimulates the user to continue to use the app
**Ease of use**		**Degree to which users believe that using the app is effortless**
	User friendly	The app is pleasant to use
	Easy to use	The app effectively fills users' needs and is fast and free of errors
	Learnability	The extent to which users are able to easily learn and understand how to operate the system
	Memorability	The extent to which users are able to remember how to use the system
**Outcome expectations**		**Congruence between users' expectations and actual situation with regard to using the app and the feedback provided by the system**
	Expectations	Users' expectations of the system
	Understanding of system	The degree of comprehension that a user possesses about the systems or services that are provided
	Confidence in the system	Users' feelings about the reliability of the app and the feedback provided by the system
	Feelings of participation	The degree of involvement and commitment which the user shares with app provider and other app users toward the functioning on system and services
	Feelings of control	Users' perceived power to regulate/ influence the feedback provided by the system
	Degree of training	The amount of specialized instruction and practice that is afforded to the user to increase the user's proficiency in utilizing the system capability that is unavailable.
	Accuracy	Users' perception that the provided feedback is congruent with their expectations about their behavior

	Health & performance effects	Users' (expected) changes in lifestyle, work performance, or other health-related issues as a result of using the App
**Organizational factors**		**Influence of the organization, procedures, and choices on the quality of the app**
	Management involvement	The positive or negative degree of interest, enthusiasm, support or participation of any management level above the users own level toward the App or towards the provider of the App
	Organizational competition	Congruence between the assessment and feedback provided by the system and an external health professional/ system (e.g. coach, other app, other system)
	Security of data	The safeguarding of data from misappropriation or unauthorized access, alteration or loss
	Documentation	The recorded description of an information system. This included formal instructions to the user and to program staff about the app
	Timing	The availability of the measurements and feedback of the app at a time suitable for its use
	Communication	The availability of correct information before using the app

References

Bailey JE, Pearson WE (1983). Development of a tool for measuring and analyzing computer user satisfaction. Management Sci, 29(5): 530-545.

Vosbergen S, Mahieu GR, Laan EK, Kraaijenhagen RA, Jaspers MWM, Peek N. (2014). Evaluating a web-based health risk assessment with tailored feedback: what does an expert focus group yield compared to a web-based end-user survey? Journal of medical internet research, 16(1): e1. DOI: 10.2196/jmir.2517.

Wixom BH, Todd PA (2005). A theoretical integration of user satisfaction and technology acceptance. Information systems research, 16(1): 85-102.
