# Supplementary material for: Evaluating an mHealth App for Health and Well-Being at Work: Mixed-Method Qualitative Study
Source: JMIR Mhealth Uhealth. 2018 Mar 28;6(3):e72. doi: 10.2196/mhealth.6335 (PMC5895922; doi:10.2196/mhealth.6335)
Supplement: Multimedia Appendix 2 [file mhealth_v6i3e72_app2.pdf]

Multimedia Appendix 2. Illustrative quotes

This is a Multimedia Appendix to a full manuscript published in the JMIR mHealth and uHealth. For full copyright and citation information see http://mhealth.jmir.org/0000/0/e0/ doi:10.2196/mhealth.6335.

Table. Illustrative examples of employees' quotes from interviews and focus groups and experts' quotes within domains and by topic with coded value (positive (+), negative (-), neutral (0) quotes or recommendation (R)).

**Domain**	**Topic**	**Illustrative quotes interviews employees**	**Illustrative quotes focus groups employees**	**Illustrative quotes focus group experts**
**System quality**	**Accessibility**	-	-	-
	**Timelines (responsiveness)**	Most of the time I check the app right before I go to sleep, because I would like to know how I did today (0)	When I receive a message, I take a look at the app. However, I take a look less often now, mostly in the evening or when I am at the toilet (0)	I looked at the app at the end of the day (0)
	**Flexibility**	I took a look at the shiftwork app. Actually I don't work in shift and did not use it, but if you travel overseas it tells you about getting rid of your jetlag, but it didn't work that well for me: it seems to start automatically, however, if you go to Belgium it seems to think that I was in another time zone (which was not the case). I think it is not that accurate (-)	I work in nightshifts, I received the advice to go cycling at 4.00 A.M. (-)	The advice has to fit with the working tasks (R)
	**Integration**	I would not try to make all functionalities in one app, but I would use apps that are already measuring that. I would choose different good apps and combine them (R).	I find retrieval of data not really easy, but I would like that (-)	I would appreciate it when the app would contain connecting services with specific wearables. Since I do not believe that a smart phone can measure everything. E.g., I put my working phone off when I quit working and a wearable is easier (R)
	**Efficiency**	When I swipe the screen, it reacts after 5 seconds (-)	It is not responsive enough, the screen hampers somewhere	-

			halfway. It feels like a 15 years ago (-)	
	**Tailoring**	There are many different kinds of workers in our company, some need physical activity advice (they lift weights a lot for instance), others have to exercise more (e.g. sitting at desk to much). App can help them to become aware. Goal of such an app is to try to get people think whether they are in balance. Do they have sufficient activity? (R)	To put things on or off, that would be convenient (R).	I believe it is important for apps: an app needs to get to know me, what my goals are, what change techniques do work, pieces of surprise (all of a sudden different tips). Towards artificial intelligence. Currently apps are often one-dimensional, it should be a learning algorithm (R)
	**Language**	-	It has a high ' hippy-level'. To fill in 'awesome' as an answer feels awkward. I do not like that. Just put things as they are (-)	English language was not of good quality (-)
	**Errors / error prevention**	-	Using the app, it's just trial and error (-)	You might give instruction in the beginning and, after a while, when a person understands the app, put them off. For instance a balloon that says "swipe to the right for…" or " did you know….". After a while these instructions disappear, once it is clear that people have found certain components or understand certain functionalities. Now it's just the screens (R)
	**Performance**	The new version was very much advanced but it was draining my battery. Not very handy going from one to another meeting and not being able to recharge phone (-)	Because of the battery use I do not use the app anymore (-)	Technically, the app feels slow (-)
**Information quality**	**Accuracy**	I did not trust the sleep measurements (-)	If the measurement is good, you also attach more value to the advices (R)	A tip was not correct: to eat repeatedly small bits of food may work for weight management, but it is not healthy from the point of view of dental care, for

				which it is really, really bad (-)
	**Precision**	If I move a lot at work, then it's measuring much. Over the weekend, it lies on my desk and measures nothing. It gives an indication and that is good enough (0)	-	-
	**Reliability**	Do I feel that measurements are true? Sometimes I have moved a lot but then I see no icon. It works only if you have your phone with you. Sometimes I do not have it with me (-)	I notice that I have reached my goal every day, but often this was not true, I have doubts about the accuracy of the app (-)	Movement measures were accurate with me (+)
	**Currency**	I read all of the articles, background information. They were not refreshed too often (-)	I have read the background information, but I find it a pity that there was no information added. Too bad this information was not being updated (-)	-
	**Completeness**	Tips are good. More exercise, better sleep, less stress. You can find a lot of information about that, for instance via the background info, you can also click further (+)	I find it special that it has different aspects together in one app (+)	Then you hope to get more information, but that is not the case. I have searched many times for information (-)
	**Format**	The motion screen is nice (+)	The design with sliding screens is OK (+)	Plots next to each other, e.g. how sleep relates to resilience, you won't see that. Also the course of resilience over time is not available (-)
	**Volume**	Annoying about it: the amount of questionnaires you get (+)	I find messages that I constantly get a bit redundant and too much (-)	Quite a lot of reading (-)
	**Content**	I would like more information about food, what you should eat. Shift workers have to eat very fast at times (and therefore, the choices are not always healthy). I would like tips about food that is healthy and that you can eat fast (R)	I would like to see more about calories in the app, I would like to add a food coach (R)	I think of an app that shows the effects of your behavior, for example to show visually: what you have done now leads to this effect (R)
	**Visibility of system status**	-	-	-

**Service quality**	**Relationship with app provider**	Technical support contact: I got personal response (+)	-	-
	**Communication with app provider**	Technical support contact: I got response but not what they had done. I would have liked to have received a message that I had to remove the app for a while (R)	There are many people who have had contact with the Brightr team (0)	-
	**Technical competence of app provider**	You can see that they constantly take further steps in developing the app	-	-
	**Attitude of app provider**	-	-	-
	**Schedule of products or services**	Technical support contact: I received a response within a day (+)	-	-
	**Processing of change requests**	-	-	-
	**Response time**	-	-	-
	**Means of input with app provider**	Right now I lost the password (after installing) and cannot find it anymore (-)	I also asked the coach, I got a good answer (+)	-
**Usefulness**	**Usefulness**	With the app, I can better recognize coming stressful times (+)	It triggers to do things better in your behavior. The fact that I saw that I pretty quickly reached my physical activity goals was good, to see that it was not a problem for me (+)	I find it useful to gain insight that I move too little, especially when I am working at home (+)
	**Relevancy**	The best parts, for me, are the shiftwork part (I work morning, evening, night shift). Since I try to follow the advices about maintaining a healthy lifestyle and working with shift hours. It helped me to keep down the stress in my body. I felt that I could focus better on the task during the daily (nightly) work (+)	The mental resilience part is doing absolutely nothing for me. I often think for what reason am I doing this? If you are doing well, it has no added value (-)	Mental resilience also triggered ... well, it yielded only frustration, I did not receive any tips (-)
	**Adherence**	I stopped using it at some time. I understand that there has to be a learning period, but there was too minimal progress in improvement of app. I prefer to have for instance 20 questions if	A community would challenge people (R)	Because there are too many problems with the app, people will throw it away quickly (-)

		that provides good feedback instead of 1 question and not relevant feedback (-)		
**Ease of use**	**User friendly**	Usability: good, clear, but I am a technical guy. Sometimes the app is unstable and I have to restart it. Sometimes it is too slow (+)	It is not really user friendly (-)	Usability is not good enough to make people use the app spontaneously, it is not clear what it aims to do (-)
	**Easy to use**	Usability? It is unclear how you need to do some settings. For example, when your goals change. Difficult to set (-)	-	-
	**Learnability**	You have to work your way through it. That is also a disadvantage (-)	To me it's all still a bit of an unexplored area. I have a phone to call people (0)	I found out only after a week that there was more than just physical activity. I swiped once accidentally and there they were: all sorts of modules!(0)
	**Memorability**	Installing and operating was pretty clear, it was also not complicated to discover and remember how the app worked, that was all very logical (+)	-	-
**Outcome expectations**	**Expectations**	Why I use such apps: it is basically extra information. If you want to improve running, you need that extra information, and an app can offer that. I hoped that Brightr would offer that as well, but it is rather basic (-)	I know by myself when I was not physically active. I would especially like to see a trend and the app pointing that out. Since it is impossible to be successful every day (R)	-
	**Understanding of system**	Then you have another screen with biorhythms, I don't know what I have to do with that, that is totally unclear to me (-)	-	-
	**Confidence in the system**	Relationship between what the app indicates and what I actually sleep was not entirely clear (-)	If the feedback is correct, then this information is nice to know. However, if the feedback is not correct, then it is no longer useful, that is a reason not to use it anymore (-)	Implementation is weak in some parts. For example mental resilience scores are not accurate. It measures whether you experience stress, but there is no link to the causes of your stress. Also, certain tips were bad in the mental resilience part (-)

	**Feelings of participation**	I expected to be able to see ‘xx% has answered this’, that you can see how you rank in comparison to your colleagues (R)	To follow each other, give each other compliments. I would like that in the Brightr app (R)	Social: compare with peers and so on, that would help to keep the app accessible (R)
	**Feelings of control**	I found it useful to be able to adjust my goals, that encouraged me to really get more exercise (+)	Especially that you can set things by yourself I find important (R)	If you can turn of the parts that you don't like, then one does not change what might be necessary from a health point of view (R)
	**Degree of training**	-	-	You would have to be able to turn on instructions at start of use. And turn it off once someone knows how the app works (R)
	**Accuracy**	I noticed on the pop-ups that cycling is not measured accurately, same with walking. For example the pop up said: "you have been 30 minutes physically active', but in reality I cycled for an hour (-)	Those graphs, that’s not quite the image I have myself (-)	-
	**Health & performance effects**	There are many different kind of workers at our company, some need physical activity advice (e.g. they lift weights a lot at work), others have to exercise more (e.g. sitting at desk to much). App can help them to become aware. Goal of such an app is to try to get people think whether they are in balance. Do they have sufficient activity. I think it is possible that an app could help to reach goals Reaching some goals must be possible (+)	An app can improve your health (+)	The goal of the app is to raise awareness (0)
**Organizational factors**	**Management involvement**	The best way to introduce an app for employees: that definitely has to go through management, from higher level management to lower management. Not via email, people get so much email that people miss such things. Via management is also a	I would not introduce the app through the management. I also don't know if my management is aware of the existence of this app. It would be interesting to know which managers use the app. If the management is actively using this app, that would help,	It matters how you introduce the app: as a corporate app or as a fun gift (0)

		signal that they encourage you and allow you to use it. They should use it also themselves, as an example. It is important to know that the management supports it (R)	this is a big challenge (R)	
	**Organizational competition**	It is useful to monitor for a company whether your employees are doing well. For example a health check. You want to know whether there are improvements with your employees, you can measure that and, if necessary, plan actions (R)	An app is a good tool within a larger program (R)	App should be a part of a bigger program, in terms of intervention. It is supportive within an intervention (R)
	**Security of data**	I realize that with the app some things are logged somewhere central. Because you can compare yourself to the rest of the organization. I mean, how would you make comparisons otherwise? But it is not that secret for me to worry about it (0)	Privacy, I have nothing to hide (0)	Within an organization, it is always the question what the organization plans to do with the data. You should be very clear on this (R)
	**Documentation**	-	A simple manual, with photos or something, in which you can search quickly. A help function in the app itself is also an option. To be able to start quickly (R)	Tips, instructions or background information consisting of lots of text will not be read (R)
	**Timing**	It should be more predictable when new articles are added. I mean not every day, but it should be predictable. You don't know when it's updated. It is OK if it is not updated very often. But if it is not updated regularly then it should be more hidden (R)	It strikes me that this app especially disturbs you when you are deeply concentrated at work (-)	-
	**Communication**	For instance, you need to inform people with posters about the existence of the app. It is also important to pay more attention to the benefits of this app. Not	I was introduced to the app via e-mail (0)	Embed the app in team sessions, embed it in a wider program (R)

		everyone would like to install the application because they do not know specific benefits for them. The benefits should be clarified (R)		
